# Left coronary button aneurysm presenting with progressive enlargement after aortic root replacement

**DOI:** 10.1186/s13019-021-01645-1

**Published:** 2021-09-06

**Authors:** Kimihiro Kobayashi, Yoshinori Kuroda, Masahiro Mizumoto, Atsushi Yamashita, Eiichi Ohba, Shingo Nakai, Tomonori Ochiai, Tetsuro Uchida

**Affiliations:** Second Department of Surgery, Faculty of Medicine, Yamagata University, 2-2-2 Iida-Nishi, Yamagata, 990-9585 Japan

**Keywords:** Aortic root replacement, Coronary button aneurysm, Coronary ostial aneurysm

## Abstract

**Background:**

Aneurysmal degeneration of the coronary button after aortic root replacement using the button technique is a rare but potentially life-threatening complication. However, the appropriate management of this complication, including the indications for conservative and surgical treatment, is still unknown.

**Case presentation:**

Here we present a 38-year-old woman who successfully underwent surgical repair of a left coronary button aneurysm using the graft interposition technique 24 years after aortic root replacement. Because follow-up computed tomography after aortic root replacement showed a progressively enlarging left coronary button aneurysm, the patient was judged an acceptable candidate for surgical treatment, considering the potential risk of aneurysmal rupture and subsequent myocardial infarction. The postoperative recovery was uneventful. The patient is doing well 1 year after the surgery.

**Conclusions:**

We believe that serial follow-up using computed tomography is mandatory for coronary button aneurysms, and surgical intervention may be considered if progressive enlargement of the aneurysm is observed, especially in younger patients.

**Supplementary Information:**

The online version contains supplementary material available at 10.1186/s13019-021-01645-1.

## Background

With the introduction of the button technique for coronary reimplantation in patients undergoing aortic root replacement (ARR) [1], the incidence of pseudoaneurysmal formation at the coronary anastomotic site has dramatically decreased. Meanwhile, aneurysmal change of the coronary button itself has been highlighted as a rare but potentially fatal complication due to the risk of aneurysmal rupture with coronary malperfusion, which may require surgical intervention [2–8]. However, the optimal management of this complication is still unclear owing to its rarity. We present a case of successful surgical repair for a left coronary artery (LCA) button aneurysm with gradual enlargement 24 years after ARR.

## Case presentation

**Fig. 1:**
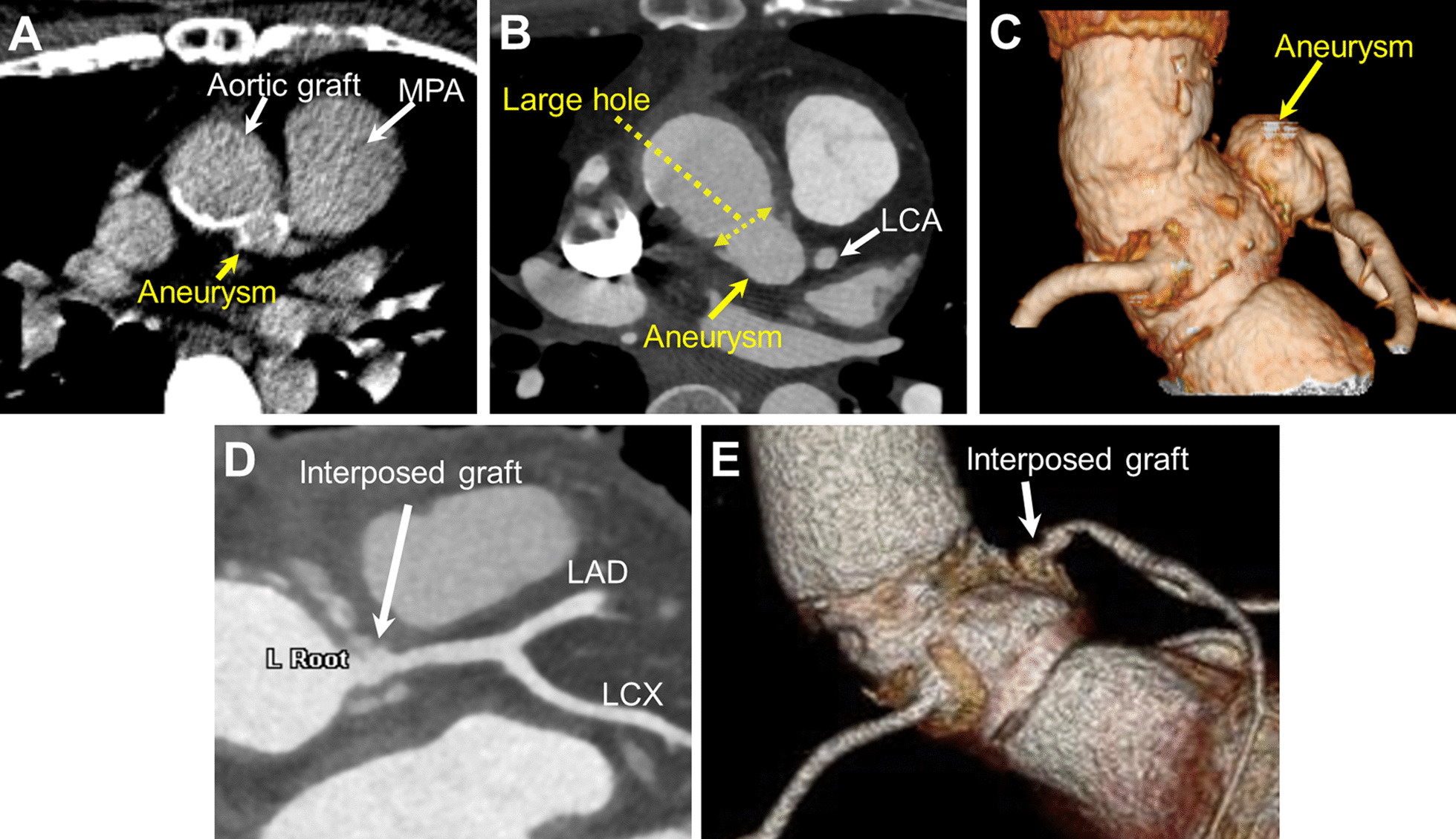
**A** Computed tomographic (CT) image 14 years after aortic root replacement shows the LCA button aneurysm with a diameter of 10 mm. **B** CT image 24 years after aortic root replacement shows that the LCA button aneurysm gradually enlarged to a diameter of 20 mm. **C** Preoperative three-dimensional reconstructed CT image shows the LCA button aneurysm. **D**, **E** Postoperative CT image shows successful reconstruction of the LCA system. LAD: left anterior descending artery, *LCA* left coronary artery, *LCX* left circumflex artery, *MPA* main pulmonary artery

Written informed consent was obtained from the patient to publish this article. A 38-year-old woman underwent valve-sparing ARR (David Procedure) with the button technique for annuloaortic ectasia at the age of 14 years. She also required mechanical aortic valve replacement for severe aortic regurgitation 1 year after the ARR. During outpatient follow-up, computed tomography (CT) revealed aneurysmal formation of the LCA button. The LCA button aneurysm gradually enlarged, growing to approximately 10 mm in 10 years to a diameter of 20 mm at the age of 38 (Fig. 1A–C). In contrast, the right coronary artery button showed no aneurysmal formation at follow-up. She did not have the typical clinical features of Marfan syndrome. Considering the potential risk of rupture and subsequent myocardial infarction due to progressive enlargement of the aneurysm, she was considered a suitable candidate for surgical repair of an LCA button aneurysm.

**Fig. 2:**
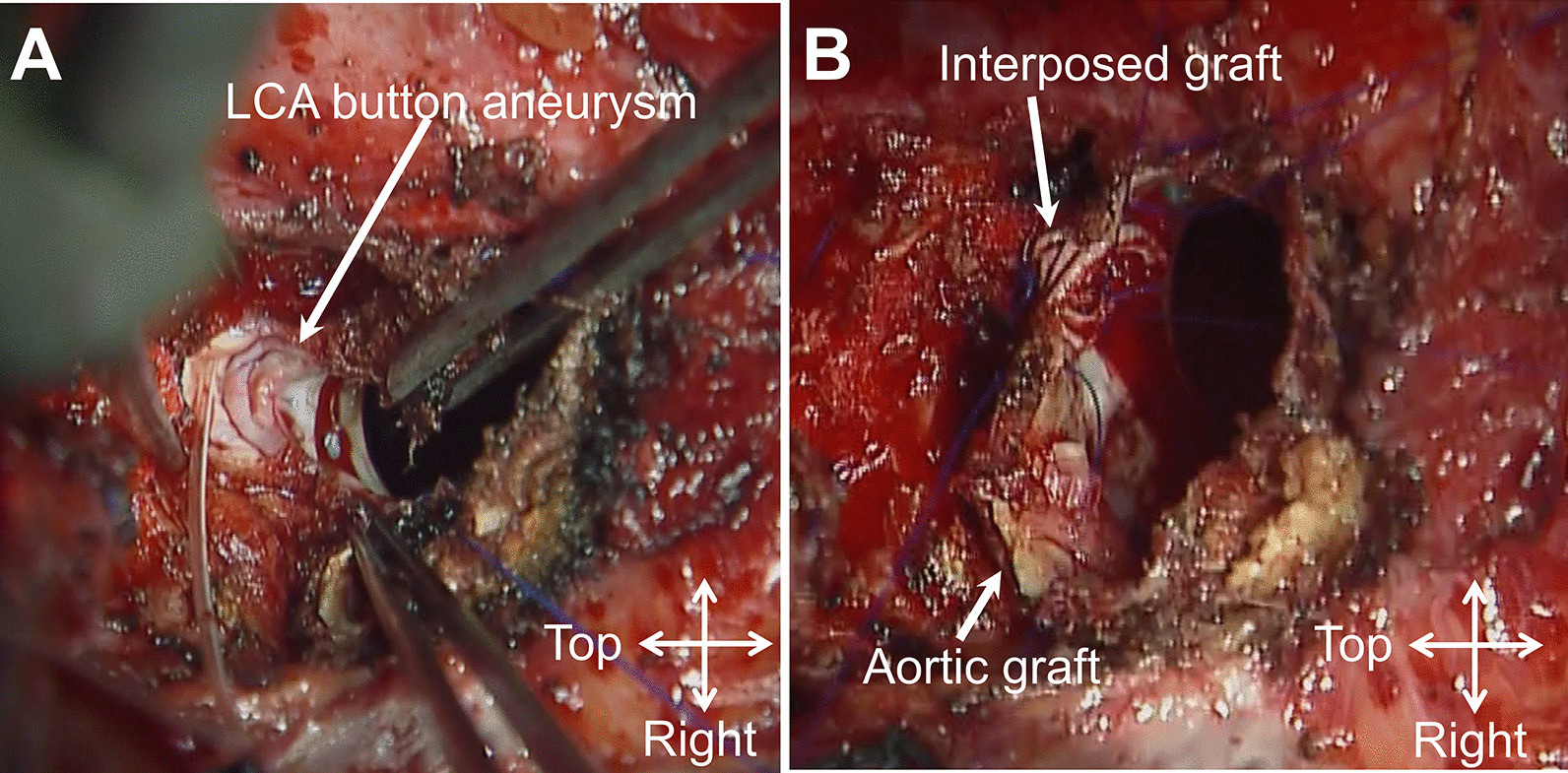
**A** Intraoperative image shows an opened LCA button aneurysm. **B** Reconstruction of LCA system using the interposition technique. *LCA* left coronary artery

Following sternal re-entry, a cardiopulmonary bypass was established using the right axillary and femoral arterial cannulation with bicaval cannulation. The previous aortic graft was transected, and the opening of the LCA button aneurysm was inspected from within the graft. The coronary hole created at the aortic graft for LCA reimplantation was large (approximately 15 mm in size). The aortic graft was incised vertically toward the aneurysmal opening to obtain surgical exposure. A new LCA button, including the aneurysmal wall, was created by isolating the aneurysm from the aortic graft. The aneurysmal wall was thin and extremely fragile. The LCA button aneurysm was gently dissected from the surrounding tissue (Fig. 2A). Considering the limited mobility of the LCA and the large coronary hole of the aortic graft, direct reimplantation of the new LCA button was considered inappropriate. Therefore, the interposition technique was employed (Fig. 2B and Additional file 1: video 1). An 8-mm prosthetic graft was anastomosed to the new LCA button using 5–0 polypropylene sutures with a large suture bite to exclude as much of the aneurysmal wall as possible and to reinforce the anastomosis. The interposed graft was trimmed appropriately and anastomosed to the aortic graft in an end-to-side fashion. Weaning from the cardiopulmonary bypass was uneventful, without bleeding or coronary malperfusion. The postoperative course was uneventful, and postoperative CT demonstrated a properly reconstructed LCA system (Fig. 1D, E).

## Discussion and conclusions

The treatment strategy for the coronary button aneurysm after ARR, whether conservative or surgical, is still unclear, because few studies have focused on the fate of the coronary anastomosis site after ARR due to its rarity. The only study that evaluated changes in the size of coronary button aneurysms after ARR using baseline postoperative and late CT data showed no significant change in mean diameter from approximately 10 mm during a mean follow-up of 5.4 years, concluding that the conditions were not progressive and of little clinical significance [9]. However, most of the reported surgical cases of coronary button aneurysms were intervened more than 10 years after ARR, and the diameters of aneurysms were mostly larger than 20 mm with the largest being 45 × 50 mm [2–6, 8]. Therefore, a 5-year follow-up period may not be enough time to evaluate changes in the size of coronary button aneurysms and late aneurysm-related events, such as rupture and dissection, and more long-term observation may be necessary. The coronary button aneurysm in our patient was unique and valuable in demonstrating a progressive growth of size over time on CT imaging, which has not been described in previous reports. If CT follow-up reveals no progressive dilation of the aneurysm, conservative treatment may be recommended, taking into account the difficulty of reoperation for this complication. However, surgical intervention may be reasonable when aneurysm is progressively enlarged, as in our case, especially in young patients.

Among several surgical options, the optimal technique for coronary button aneurysms has not been defined. Redo-ARR, exclusion of the aneurysm in combination with coronary artery bypass grafting, and in-situ anatomical reconstruction of the coronary system have previously been reported [2–5]. Redo-ARR is considered too invasive in this setting. Considering that this particular complication is more frequent in younger patients [2–8], the long-term patency of the coronary artery bypass graft may be a major problem. Anatomical coronary reconstruction using the direct anastomosis or the interposition technique is ideal and has two major technical concerns: (1) limited mobility of the coronary artery due to dense adhesions; and (2) coronary hole size of the previous aortic graft. Direct anastomosis requires extensive dissection and mobilization of the coronary artery, which can lead to injury of the surrounding structures and of the coronary artery itself in the setting of reoperation. In contrast, the interposition technique allows tension-free anastomosis with minimal dissection, which may be effective in preventing bleeding due to coronary overstretching and coronary malperfusion due to kinking. Furthermore, in this complication, the size of the coronary hole in the previous aortic graft is very large. Therefore, direct anastomosis between the large coronary hole and the native coronary ostium is considered technically difficult because of the size discrepancy. Meanwhile, the interposed graft can be flexibly trimmed to fit the large coronary hole. Considering these advantages of the interposition technique, we believe that this technique is safer and more reliable for this challenging complication.

In conclusion, cardiovascular surgeons should bear in mind that long-term follow-up CT after ARR for more than 10 years should be performed to check for aneurysmal formation at the button sites and increase in aneurysm size. Additionally, if progressive enlargement of the coronary button aneurysm is observed, surgical intervention should be considered, taking into account the patient's comorbidities and age.

## Supplementary Information

**Additional file 1: Video 1**. Reconstruction of the left coronary button aneurysm using the interposition technique.

## Data Availability

The data are not available for public access due to patient privacy concerns but are available from the corresponding author upon reasonable request.

## Abbreviations

ARR: Aortic root replacement
CT: Computed tomography
LCA: Left coronary artery
